# PET/MR synchronization by detection of switching gradients

**DOI:** 10.1186/2197-7364-1-S1-A4

**Published:** 2014-07-29

**Authors:** Bjoern Weissler, Pierre Gebhardt, Christoph W Lerche, Georgios Soultanidis, Jakob Wehner, Dirk Heberling, Volkmar Schulz

**Affiliations:** Department Molecular Imaging Systems, Philips Research Europe - Aachen, Pauwelsstrasse 17, D-52074 Aachen, Germany; Department of Physics of Molecular Imaging Systems, Institute of Experimental Molecular Imaging, RWTH Aachen University, Pauwelsstrasse 20, D-52074 Aachen, Germany; Institute of High Frequency Technology, RWTH Aachen University, Melatener Straße 25, D-52074 Aachen, Germany; Imaging Sciences & Biomedical Engineering, King’s College London, St Thomas Hospital, London, SE1 7EH UK

The full potential of simultaneous PET and MRI image acquisition, such as dynamic studies or motion compensation, can only be explored if the data of both modalities are temporally synchronized. These hybrid imaging systems are often realized as custom made PET inserts for commercially available MRI scanner. Unfortunately, the standard MRIs do not always offer easily programmable synchronization outputs, nor can they be modified.

Here, we demonstrate a simple method for temporal synchronization that does not require a connection to the MRI. It uses the normally undesired effect of induced voltages on the PET electronics by switching MRI gradients.

In our preclinical PET/RF inserts the PET detector electronics are located in RF-tight housings between the gradient coil and the RF coil. Unlike RF fields, gradient fields penetrate the housings due to their relatively low frequencies. A dedicated coil system was made from traces and vias on the PET detector board. It captures induced voltages from the switching gradients and a time-stamped gradient trigger message is added to the PET data stream.

Temporal alignment between PET and MRI data can be made at the beginning of each MRI sequence by detecting its first gradient or by recognizing its preparation phase. From then on, the MRI time can be translated to the PET time.

Detecting the preparation phase of an MRI sequence was tested with a standard survey sequence (for brain) using the PET/RF insert “Hyperion I” inside a Philips 3T Achieva MRI. The three crusher gradient pulses (slew rate = 25 mT/m/ms) used during noise level determination can clearly be recognized in the data from the sensors.

As a first application the sensors were used to synchronize PET and MRI time to facilitate MRI based PET motion compensation of a deforming breathing phantom.

